# Supplementary material for: Meta-analytical approaches to ordinal outcome data in clinical interventional studies: A scoping review with reproducible research
Source: PLoS One. 2025 Apr 1;20(4):e0313720. doi: 10.1371/journal.pone.0313720 (PMC11960913; doi:10.1371/journal.pone.0313720)
Supplement: Attachment — Submitted filename: Response to reviewers.docx [file pone.0313720.s003.docx]

**A point-by-point response to reviewers**

• **Have the authors made all data underlying the findings in their manuscript fully available?**
**Reviewer #1:** No
**Response:** Additional data have now been made available.

• **Background information is insufficient, limiting a robust justification for why this research is needed and what it will add to the existing knowledge.**
**Response:** A detailed section has been added to the introduction, providing a stronger justification for the research. It highlights the gaps in current knowledge, the importance of addressing these gaps, and the contributions this study makes to the field.

• **Methods: Can you explicitly state the framework used to guide the scoping review in the methods section?**
**Response:** The PRISMA framework has been explicitly mentioned under the “Scoping Review/Protocol and Registration” subsection to clarify the methodological guidance used.

• **Methods: What specific approaches or thinking fed into the reproducible analysis?**
**Response:** The primary aim and methodological approach of the reproducible analysis have been clarified in the “Reproducible Research” subsection of the Methods. We specified the rationale for verifying prior findings and addressing discrepancies.

• **Results: The paragraph explaining the screening process is unclear; how did you arrive at the 108 papers, of which 104 were excluded?**
**Response:** The paragraph describing the screening process has been rewritten for clarity. It now includes detailed steps on how the records were screened, excluded, and the reasons for exclusions.

• **Results: Where are the included studies from, and which interventions did they focus on? Were the interventions similar across the included studies?**
**Response:** The included studies are methodological studies focused on meta-analytical approaches for ordinal outcome data. As such, they do not address specific interventions but rather examine statistical methods. This is consistent with the stated eligibility criteria.

• **Table 1, which is supposed to show the characteristics of the included studies, cannot be found in the manuscript or in any supporting file.**
**Response:** Table 1 has now been included in the revised manuscript and supporting documents. We apologize for its omission in the initial submission.

• **Were the authors (those who provided the raw data) contacted regarding the discrepancies in the findings? If yes, what explanations were generated?**
**Response:** Yes, the authors were contacted. A detailed clarifying paragraph has been added under “Reproducible Research Analysis” in the Results section to address the discrepancies and the steps taken to resolve them.

• **What specific and critical research implications does your study offer for conducting meta-analysis with ordinal outcomes?**
**Response:** The critical implications have been elaborated on in the revised Conclusion and Implications section. This includes guidance on choosing statistical methods and the need for methodological rigor and reproducibility in future research.
